# Pulmonary Sporotrichosis Caused by *Sporothrix brasiliensis*: A 22-Year, Single-Center, Retrospective Cohort Study

**DOI:** 10.3390/jof8050536

**Published:** 2022-05-21

**Authors:** Vivian Fichman, Caroline Graça Mota-Damasceno, Anna Carolina Procópio-Azevedo, Fernando Almeida-Silva, Priscila Marques de Macedo, Denise Machado Medeiros, Guis Saint-Martin Astacio, Rosely Maria Zancopé-Oliveira, Rodrigo Almeida-Paes, Dayvison Francis Saraiva Freitas, Maria Clara Gutierrez-Galhardo

**Affiliations:** 1 Laboratory of Clinical Research in Infectious Dermatology, Evandro Chagas National Institute of Infectious Diseases (INI), Oswaldo Cruz Foundation (FIOCRUZ), Rio de Janeiro 21040-360, RJ, Brazil; vivianfichman@gmail.com (V.F.); carolgracamd1@gmail.com (C.G.M.-D.); priscila.marques@ini.fiocruz.br (P.M.d.M.); maria.clara@ini.fiocruz.br (M.C.G.-G.); 2 Laboratory of Mycology, Evandro Chagas National Institute of Infectious Diseases (INI), Oswaldo Cruz. Foundation (FIOCRUZ), Rio de Janeiro 21040-360, RJ, Brazil; anna.azevedo@ini.fiocruz.br (A.C.P.-A.); fernando.almeida@ini.fiocruz.br (F.A.-S.); rosely.zancope@ini.fiocruz.br (R.M.Z.-O.); rodrigo.paes@ini.fiocruz.br (R.A.-P.); 3 Medical Service, Evandro Chagas National Institute of Infectious Diseases (INI), Oswaldo Cruz Foundation (FIOCRUZ), Rio de Janeiro 21040-360, RJ, Brazil; denise.medeiros@ini.fiocruz.br; 4 Image Service, Evandro Chagas National Institute of Infectious Diseases (INI), Oswaldo Cruz Foundation (FIOCRUZ), Rio de Janeiro 21040-360, RJ, Brazil; guis.sma@gmail.com

**Keywords:** pulmonary sporotrichosis, *Sporothrix brasiliensis*, zoonotic transmission, AIDS

## Abstract

Pulmonary sporotrichosis is a rare condition. It can present as a primary pulmonary disease, resulting from direct *Sporothrix* species (spp). conidia inhalation, or as part of multifocal sporotrichosis with multiple organ involvement, mainly in immunocompromised patients. This study aimed to describe the sociodemographic and epidemiological characteristics and clinical course of patients with positive cultures for *Sporothrix* spp. from pulmonary specimens (sputum and/or bronchoalveolar lavage) at a reference center in an area hyperendemic for zoonotic sporotrichosis. The clinical records of these patients were reviewed. Fourteen patients were included, and *Sporothrix brasiliensis* was identified in all cases. Disseminated sporotrichosis was the clinical presentation in 92.9% of cases, and primary pulmonary sporotrichosis accounted for 7.1%. Comorbidities included human immunodeficiency virus infection (78.6%), alcoholism (71.4%), and chronic obstructive pulmonary disease (14.3%). Treatment with amphotericin B followed by itraconazole was the preferred regimen and was prescribed in 92.9% of cases. Sporotrichosis-related death occurred in 42.9% while 35.7% of patients were cured. In five cases there was a probable contamination from upper airway lesions. Despite the significant increase in sporotrichosis cases, pulmonary sporotrichosis remains rare. The treatment of disseminated sporotrichosis is typically difficult. Prompt diagnosis and identification of all affected organs are crucial for better prognosis.

## 1. Introduction

Sporotrichosis is a subcutaneous mycosis with worldwide distribution, especially in tropical and subtropical areas, and is caused by dimorphic fungi of the genus *Sporothrix* [1]. The classical infection route is associated with traumatic inoculation of subcutaneous tissues by the etiological agent while manipulating soil or other organic materials containing the conidia. Zoonotic transmission, especially from naturally infected cats, and fungal conidial inhalation from the environment are other routes of infection [2].

In the past two decades, the state of Rio de Janeiro, Brazil, has emerged as a hyperendemic region of zoonotic sporotrichosis, transmitted by cats and associated with *Sporothrix brasiliensis*, the most virulent species of the genus [3,4]. In addition to benign cutaneous forms, other rare forms of this mycosis have been observed, affecting the bones, mucosa, and central nervous system [5,6,7]. Endemic areas of zoonotic sporotrichosis, along with these unusual manifestations of the disease, have spread throughout South America in recent years [8].

Pulmonary sporotrichosis is a rare manifestation, presenting with two clinical patterns: primary pulmonary sporotrichosis (PPS), a unifocal disease resulting from direct fungal inhalation, and multifocal sporotrichosis, when the fungus is acquired by direct inhalation of conidia with subsequent dissemination or, more commonly, by hematogenous or lymphatic spread from a distal site due to immunosuppression. While patients with PPS tend to have underlying respiratory conditions (mainly chronic obstructive pulmonary disease (COPD)), those with the multifocal form are mostly immunosuppressed [9]. The respiratory clinical presentation is nonspecific, similar to that of tuberculosis (TB) and endemic systemic mycoses. In PPS, the radiological pattern is cavitary, while in multifocal disease, it is non-cavitary with multilobar distribution [10].

The treatment of both conditions is difficult. In mild to moderate lung disease, itraconazole is administered orally at 200 mg twice daily for 12 months. In severe disease, a lipid formulation (lipid complex or liposomal) of amphotericin B 3–5 mg/kg is recommended until a favorable response is achieved; subsequently, therapy can be changed to itraconazole 200 mg twice daily for a total of 12 months. Surgery combined with amphotericin B therapy is recommended for localized pulmonary disease [11].

In 2019, Falcão et al. [12] analyzed data from the Brazilian Unified National Health System (SUS) and reported pulmonary sporotrichosis to be the leading clinical form of the disease among hospitalized patients. This form accounted for at least 220 hospitalizations, corresponding to 35.9% of hospitalized cases of sporotrichosis registered in Brazil from 1998 to 2015.

Because sporotrichosis is hyperendemic in Rio de Janeiro, we studied the impact of pulmonary sporotrichosis in our region. More specifically, this study aimed to describe the sociodemographic and epidemiological characteristics and clinical course of patients with pulmonary specimens positive for *Sporothrix* species (spp.) at a reference center for sporotrichosis in Rio de Janeiro, Brazil.

## 2. Materials and Methods

### 2.1. Study Design

This study was approved by the Research Ethics Committee of Instituto Nacional de Infectologia Evandro Chagas (INI/FIOCRUZ), RJ, Brazil (appreciation number 08097112.3.0000.5262). A retrospective observational study, involving a cohort of patients diagnosed with sporotrichosis between 1998 and 2019, was conducted at the INI/FIOCRUZ. The inclusion criterion was the presence of a positive culture for *Sporothrix* spp. obtained from pulmonary specimens (sputum and/or bronchoalveolar lavage (BAL)). The records of the Mycology Laboratory were reviewed for positive *Sporothrix* cultures. The strains were stored in the Collection of Pathogenic Fungi at the Mycology Laboratory of INI.

### 2.2. Clinical Management

Briefly, patients with suspected sporotrichosis underwent standard clinical and laboratory evaluation on admission. Skin samples or samples from other clinically suspicious sites (biopsy, exudate, or aspirate) were collected for fungal culture. In cases of suspected disseminated disease or in patients with known immunosuppression, other samples, such as sputum, blood, cerebrospinal fluid, and urine were collected for routine mycological examination, as well as imaging studies, as previously described [2,13,14]. After the first positive sputum culture for *Sporothrix* spp., serial sputum cultures were collected until patients were considered to be cured and discharged, particularly in the case of PPS. The protocol also included microbiological and serological tests for the differential diagnosis of major endemic pulmonary diseases, such as TB, paracoccidioidomycosis, and histoplasmosis.

### 2.3. Laboratory Methods

Cultures of clinical specimens were made on BBL Sabouraud dextrose agar 2% (Becton Dickinson Co., Sparks, MA, USA) and Mycosel Agar (Becton Dickinson Co., Sparks, MA, USA) and incubated at 25 °C for up to four weeks. Dimorphism of suspected *Sporothrix* colonies was assessed on brain heart infusion agar (Becton Dickinson Co., Sparks, MA, USA) at 35 °C for seven days. Molecular identification of pure and viable *Sporothrix* spp. strains isolated from the included patients was performed using a species-specific polymerase chain reaction protocol [15].

### 2.4. Treatment

Severe cases were treated with amphotericin B until clinical improvement, followed by itraconazole. In less severe cases, oral itraconazole 200 mg was administered twice daily for at least 12 months [11]. In some cases of partial response to itraconazole, terbinafine was added (or replaced itraconazole) based on the previous experience of the group treating cutaneous forms [16]. Since 2013, posaconazole (800 mg/day) has been the oral antifungal agent of choice for refractory cases of sporotrichosis at the authors’ institution [17].

### 2.5. Data Analyses

Patient medical records were reviewed to collect sociodemographic, epidemiological, clinical, radiological, and follow-up data. Data were anonymized and de-identified to protect patient privacy and confidentiality. The data obtained were stored in a spreadsheet (Excel, version 2016, Microsoft Corporation, Redmond, WA, USA), and the statistical program R-Project version 4.1.0 (R Core Team (2021), Vienna, Austria, https://www.R-project.org/, accessed on 29 April 2022) was used for descriptive analyses, such as frequencies for categorical variables and summary measures (mean, median, and range) for continuous variables.

### 2.6. Literature Search

To identify published cases of sporotrichosis in Brazil, a Medline search was performed using the keywords “zoonotic”, “sporotrichosis”, “pulmonary”, “lung”, and “Brazil”.

## 3. Results

### 3.1. Laboratory Data

Between 1998 and 2019, 5264 patients were diagnosed with sporotrichosis at INI/FIOCRUZ. Fourteen (0.27%) patients had positive cultures from pulmonary specimens, totaling 17 samples. There were 13 sputum samples (seven induced sputum samples) and four positive BAL samples. *S. brasiliensis* was identified as the causative species in all 14 cases. Serological tests for the detection of anti-*Paracoccidioides* and anti-*Histoplasma* antibodies were negative in all patients.

### 3.2. Patient Data

Demographic, epidemiological, clinical, and follow-up data from the patients are summarized in Table 1. Males accounted for 78.6% (*n* = 11) of cases. The mean age of the patients was 37.9 years, 78.6% (*n* = 11) were non-white, and 21.4% (*n* = 3) were white. The median number of years of schooling was 6 (range, 0–13 years). Regarding occupation, 71.4% (*n* = 10) had no formal work activity, including unemployed status (*n* = 8), retired (*n* = 1), or student (*n* = 1). The income of 64.3% (*n* = 9) of patients was lower than the national minimum wage. All but one resided in Rio de Janeiro or neighboring municipalities. Contact with cats was reported by 71.4% of patients (*n* = 10) and, within this group, 90.0% (*n* = 9) reported that the cat was sick; bites or scratches were reported by 44.4% (*n* = 4) of these nine patients. Contact with soil was reported by 35.7% (*n* = 5). Two patients had contact with cats, soil, or plants. No possible source of infection was identified in one patient.

**Figure 1:**
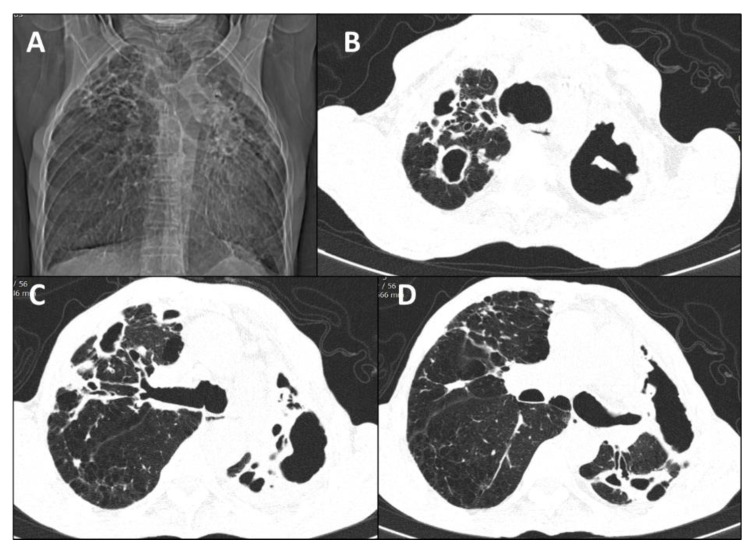
Primary pulmonary sporotrichosis in a 71-year-old man (Case 1). (**A**) Topogram depicts lung cavitary lesions. (**B**–**D**) Axial nonenhanced chest computed tomography images show extensive thick-walled cavities with irregular margins in upper lobes. The cavitary lesion is more predominant in the left upper lobe, associated with thick septations and pulmonary volume loss.

**Figure 2:**
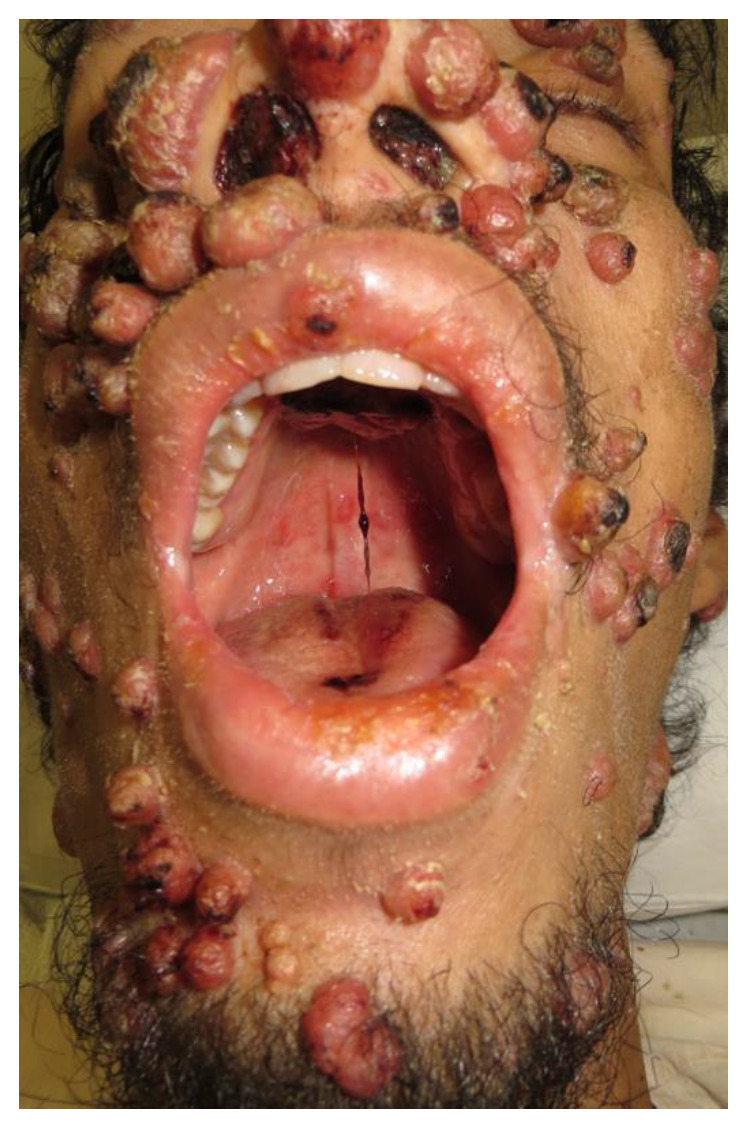
Disseminated sporotrichosis in a 25-year-old man (Case 4). Multiple papular and nodular-ulcerative facial lesions, with aerodigestive tract impairment. Involvement of the nasal mucosa and palate is shown.

Disseminated sporotrichosis was the clinical presentation in 92.9% (*n* = 13) of cases, with PPS accounting for 7.1% (*n* = 1) of cases (Figure 1). In the disseminated forms, the most involved organs and systems were the skin (85.7% (*n* = 12)) and bones (85.7% (*n* = 12)), followed by the lungs (64.3% (*n* = 9)) and upper airways (57.1% (*n* = 8)) (Figure 2).

All patients had immunosuppressive conditions or other comorbidities, including human immunodeficiency virus (HIV) infection (78.6% (*n* = 11)), alcoholism (71.4% (*n* = 10)), a combination of both (50% (*n* = 7)), and COPD (14.3% (*n* = 2)). Among people living with HIV (PLHIV), the median CD4-positive (CD4+) T lymphocyte count at the time of the isolation of *Sporothrix* spp. from the pulmonary specimen was 56 cells/mm^3^ (range, 21–178 cells/mm^3^). Smoking was reported in 78.6% (*n* = 11) of patients.

Signs and symptoms included malaise (100% (*n* = 14)), weight loss (85.7% (*n* = 12)), fever (78.6% (*n* = 11)), productive cough (64.3% (*n* = 9)), hemoptysis (14.3% (*n* = 2)), and dyspnea (7.1% (*n* = 1)).

**Figure 3:**
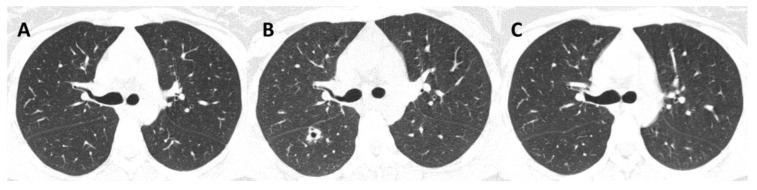
Nonenhanced chest computed tomography (CT) of a 20-year-old woman with disseminated sporotrichosis and lung lesions, at three time points (Case 14). (**A**) During treatment for confirmed pulmonary tuberculosis, CT shows no lung cavities. (**B**) Post-treatment for tuberculosis and recent diagnosis of sporotrichosis, CT shows a thick-walled cavity in the right upper lobe, with isolation of *Sporothrix* spp. from sputum. (**C**) Resolution of the cavity during sporotrichosis treatment.

Aside from HIV, other co-infections were found in 57.1% (*n* = 8) of patients, with TB the most common (*n* = 4). One patient with hemoptysis had a TB coinfection and one was previously treated for confirmed TB (Figure 3).

### 3.3. Radiological Patterns

**Figure 4:**
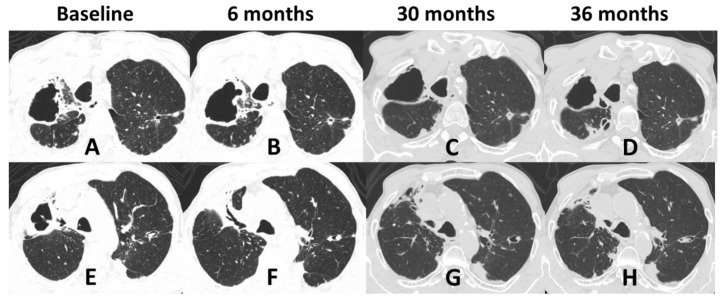
Nonenhanced chest computed tomography of a 63-year-old man with disseminated sporotrichosis and lung lesions, at four moments (Case 2). (**A**,**E**) At the beginning of treatment for sporotrichosis. (**B**,**F**) Six months of treatment. (**C**,**G**) At 30 months of treatment. (**D**,**H**) At 36 months of treatment. Among many alterations, there is a thick-walled cavity with irregular margins in the right lower lobe, associated with architectural distortion, fibrotic opacities, and traction bronchiectasis. There is also bronchiectasis in the apicoposterior segment of the left upper lobe, some filled by fluid density material, suggestive of mucous plugging. Areas of pleural thickening in the upper third of the lungs. Images A to D and E to H are similar sections over time.

Regarding radiological patterns observed, non-cavitary disease was present in 35.7% (*n* = 5) and cavitary disease was present in 21.4% (*n* = 3) (Figure 1, Figure 3 and Figure 4). No alterations were found in the imaging examinations of 42.9% (*n* = 6) of the patients, among whom, 83.3% (*n* = 5) had upper airway involvement of sporotrichosis.

### 3.4. Treatment Response and Outcomes

The median time between symptom onset and the start of treatment was 93.5 days (range, 38–467 days). Treatment with amphotericin B, followed by itraconazole, was the treatment of choice, performed in 92.9% (*n* = 13) of cases. Within this group, at some point in the disease course, two patients also received terbinafine, two received posaconazole, and three received both. In case 1, the patient with PPS with no dissemination was treated with itraconazole 200–400 mg/day alternating with posaconazole due to a poor response (Table 1). The median treatment time was 1044 days (range, 239–3115 days).

By the end of the study, 42.9% (*n* = 6) of patients died due to sporotrichosis complications, 35.7% (*n* = 5) were cured, 14.3% (*n* = 2) were still undergoing treatment, and 7.1% (*n* = 1) were lost to follow-up. All patients who died had disseminated sporotrichosis and AIDS as the underlying causes of death, and four also had TB coinfection.

### 3.5. Brazilian Cases of Pulmonary Sporotrichosis from Zoonotic Transmission

Four case reports were of disseminated sporotrichosis, two had a history of alcohol abuse, and two had HIV infection. The outcomes were cure in three cases and death in one [18,19,20,21]. The fifth report was a fatal case of PPS in an immunocompetent and non-smoker female [22]. She presented with multiple bilateral pulmonary nodules, some with central cavitation and without cutaneous lesions. In two of these cases, *S. brasiliensis* was isolated from sputum [19] and BAL [22].

## 4. Discussion

In this study, the number of cases with pulmonary specimens positive for *Sporothrix* spp. represented 0.27% of the sporotrichosis cases treated at INI/FIOCRUZ. Unlike most dimorphic fungal pathogens that cause respiratory disease in mammals [23], *Sporothrix* is the only fungal agent in which the lung is not the main route of infection; therefore, pulmonary sporotrichosis is not prevalent. In the first large sporotrichosis epidemic, described from mines in Transvaal, South Africa, 3000 cases of the cutaneous form were reported, and no cases of pulmonary sporotrichosis were diagnosed [24]. Experimentally, in mice, pulmonary infection was achieved through intranasal inoculation with *Sporothrix* spp. [25]. However, this did not occur via inhalation in an environment with a high density of conidia [26].

The emergence of hyperendemic zoonotic sporotrichosis in Rio de Janeiro is accompanied by a different and more severe clinical profile [3,27]. Arinelli et al. [28] described a series of 120 cases of ocular sporotrichosis that had been attended to over a period of 10 years. The authors highlighted the increase in the frequency of this clinical form and the risk for ocular infection through aerosols formed when cats with sporotrichosis sneeze. Cats present a high fungal load and respiratory tract signs are the most frequent extracutaneous sign [29,30]. Interestingly, inhalation of *Sporothrix* in this context of zoonotic transmission was not accompanied by an increase in PPS cases. In a Medline search for pulmonary involvement in zoonotic sporotrichosis from Brazil, few cases have been described, with a profile similar to that in our study. Although rare, pulmonary sporotrichosis can be underestimated and misdiagnosed as other pulmonary infections, such as bacterial pneumonia and TB, due to its nonspecific clinical picture, especially in cases of PPS.

The sample population in this study was mostly male, with low educational and economic status. Multifocal disease was predominant, with disseminated skin lesions, and the majority were PLHIV. The overlap of sporotrichosis with the HIV pandemic could account for this finding because our center is a reference facility for both infections. In addition, CD4+ T cells play a pivotal role in the control of sporotrichosis, and these cells are the main target of HIV infection [13,31]. In this study, all PLHIV had low CD4+ T cell counts and there was not a clear correlation between low CD4+ T cell counts and the outcome of sporotrichosis. As this was a small sample, it was difficult to compare those who were cured versus those who died. However, patients who died had more disseminated disease, especially in the central nervous system. In addition to HIV coinfection, alcoholism is another commonly known immunosuppressive condition present among our patients. There is a high prevalence of alcohol abuse in the Brazilian population, estimated to be as high as 13.7% [32]. Chronic alcohol abuse results in lymphopenia and chronic activation of the T cell pool, which may negatively impact the ability of T cells to expand and respond to pathogenic agents, inducing an anergic state and altering T-helper (Th)1 and Th2 responses [33]. Furthermore, the species involved in all the cases described herein, *S. brasiliensis*, is implicated in more severe and atypical forms of sporotrichosis [6,27].

The symptoms presented were nonspecific and the diagnosis of pulmonary involvement was often identified by routine screening. In two cases (Cases 1 and 2), the most likely transmission route was conidial inhalation. Both patients were male, smokers, and had COPD, fitting the risk profile previously reported, in which PPS most often presents in males with structurally abnormal lungs [9]. The radiological pattern that presents with cavitary disease is also described as typical of PPS [9,34]. Case 2 also had osteoarticular involvement, and the fungus may have spread from the lungs to the bones by hematogenous dissemination because the patient experienced no previous cutaneous trauma history or skin lesions.

In a systematic review of 86 cases of pulmonary sporotrichosis, 64 (74.4%) were PPS [9], 76 (88.4%) were from the United States, and only two were from Brazil (2.3%). Recently, a study using a large commercial health insurance database in the United States from 2012 to 2018 identified 1322 sporotrichosis cases [35]. Among the cases in which sporotrichosis forms were specified, lymphocutaneous was the most common (68 cases), followed by pulmonary sporotrichosis (27 cases).

In the present study, nine patients had pulmonary sporotrichosis and five patients with disseminated disease (Cases 8 to 12) had *S. brasiliensis* isolated from sputum with no pulmonary disease. These five patients exhibited sporotrichosis lesions in the upper airways, and contamination of the sputum during collection of the clinical sample is the most plausible explanation for *S. brasiliensis* isolation from sputum in these cases. In 1978, Lowenstein et al. [36] postulated the presence of *Sporothrix schenckii* as a pulmonary saprophyte. They described an asymptomatic patient with four serial sputum cultures with *Sporothrix* growth and no evidence of *Mycobacterium tuberculosis* infection. His chest radiography revealed the presence of bilateral apical cavities. In our opinion, based on the literature and on the present study, this patient might have had PPS. Evidence to declare *Sporothrix* spp. a pulmonary saprophyte is not yet well-established.

Considering the therapeutic response, all patients except one, underwent combined treatment with amphotericin B, with a low cure rate and high lethality. Despite long-term antifungal treatment, especially in patients with PPS (Cases 1 and 2), sterilization of sputum is challenging. Their systemic and respiratory conditions deteriorate, with significant weight loss and muscle wasting, which makes surgical intervention exceedingly difficult, if not futile. Extensive necrosis and fibrosis in cavitary disease could impair antifungal tissue penetration, and some authors advocate that surgical resection combined with antifungal therapy (pre- or post-surgery) may offer a better chance of cure than medical treatment alone, provided that the patient has adequate clinical conditions to survive such an intervention [37]. In the present study, respiratory involvement was present in the disseminated form; however, most prominent were the conditions of a systemic disease in an immunosuppression scenario, in which clinical deterioration and death were not related to the lung itself.

## 5. Conclusions

Despite the significant increase in the number of sporotrichosis cases in Rio de Janeiro, Brazil, pulmonary sporotrichosis remains rare. In areas endemic for TB and sporotrichosis, in respiratory cases with negative TB tests, routine investigation of *Sporothrix* spp., along with other dimorphic fungi, should be performed. For pulmonary localized lesions with limited response to antifungal agents, segmental resection should be considered early to avoid missing the opportunity for a favorable preoperative clinical condition. In cases of multifocal disease, extra caution should be exercised when diagnosing pulmonary sporotrichosis through positive sputum. Therefore, it is necessary to exclude contamination from the upper airway lesions. The treatment of disseminated sporotrichosis is typically difficult. Prompt diagnosis and identification of all affected organs are crucial for better prognosis.

## Figures and Tables

**Table 1:** Clinical data of patients with positive cultures from pulmonary specimens at the Evandro Chagas National Institute of Infectious Diseases, from 1998 through 2019.

Case (Year) ^1^	Sex	Age	Risk Exposure	Clinical Presentation	Organs and Systems Affected	Comorbidities/ Immunosuppression ^2^	Other Coinfections	Radiological Findings	Pulmonary Positive Culture Specimen	Treatment	Outcome
**1** **(2018)**	Male	71	None	Primary pulmonary	Lungs	Alcoholism COPD	No	Reticulonodular infiltrate; cavitation; fibrosis Unilateral/Multilobar	Sputum; BAL	ITZ; PSZ	Under treatment
**2** **(2018)**	Male	63	Contact with cat	Disseminated	Lungs, bone	Alcoholism COPD	No	Cavitation; fibrosis; hilar lymphadenopathy Unilateral/Unilobar	Sputum; BAL	ITZ; AmB; PSZ	Under treatment
**3** **(2015)**	Male	52	Contact with soil/plants	Disseminated	Skin, lungs, bone	HIV (CD4 = 46 cells/mm^3^)	Sepsis caused by *Klebsiella*	Nodule without cavitation Unilateral/Unilobar	BAL	ITZ; AmB	Cure
**4** **(2012)**	Male	25	Contact with soil/plants	Disseminated	Skin, lungs, bones, upper airways, eyes	HIV (CD4 = 25 cells/mm^3^) Alcoholism	Tuberculosis	Multiple nodules without cavitation Bilateral/Mulilobar	Sputum; BAL	ITZ; TBF; AmB; PSZ	Death
**5** **(2015)**	Female	31	Contact with diseased cats	Disseminated	Skin, bones	HIV (CD4 = 21 cells/mm^3^)	Pulmonary sepsis caused by *Acinetobacter* Kaposi’s sarcoma	None	Sputum	ITZ; AmB	Cure
**6** **(2012)**	Female	20	Scratched by diseased cat	Disseminated	Skin, bones, CNS	HIV (CD4 = 42 cells/mm^3^)	No	Multiple Nodules without cavitation Bilateral/Multilobar	Sputum	ITZ; TBF; AmB; PSZ	Death
**7** **(2007)**	Male	44	Contact with soil/plants	Disseminated	Skin, bone, CNS	HIV CD4 = 110 cells/mm^3^) Alcoholism	Tuberculosis	Pleural effusion	Sputum	ITZ; AmB	Death
**8** **(2008)**	Male	26	Contact with diseased cat	Disseminated	Skin, upper airways, CNS	HIV (CD4 = 178 cells/mm^3^) Alcoholism	No	None	Sputum	ITZ; AmB	Death
**9** **(2011)**	Male	36	Scratched by diseased cat	Disseminated	Skin, bones, upper airways	HIV (CD4 = 66 cells/mm^3^)	No	None	Sputum	ITZ; TBF; AmB	Loss of follow-up
**10** **(2019)**	Male	46	Bitten by diseased cat	Disseminated	Skin, bones, upper airways	HIV (CD4 = 35 cells/mm^3^) Alcoholism	No	None	Sputum	ITZ; AmB	Cure
**11** **(2003)**	Male	18	Contact with diseased cat and with soil/plants	Disseminated	Skin, bones, upper airways, eyes	Alcoholism	Nocardiosis	Non	Sputum	ITZ; TBF; AmB	Cure
**12** **(2017)**	Male	35	Contact with diseased cat (sneezing) and with soil/plants	Disseminated	Skin, bones, upper airways, CNS, eyes	HIV (CD4 = 75 cells/mm^3^) Alcoholism	Tuberculosis	None	Sputum	ITZ; AmB	Death
**13** **(2013)**	Male	43	Scratched by diseased cat	Disseminated	Skin, bones, upper airways	HIV (CD4 = 35 cells/mm^3^) Alcoholism	Tuberculosis	Diffuse reticulonodular infiltrate; calcified nodules, fibrosis	Sputum	ITZ; TBF; AmB; PSZ	Death
**14** **(2018)**	Female	20	Contact with diseased cats	Disseminated	Skin, lungs, bones, upper airways	HIV (CD4 = 56 cells/mm^3^) Alcoholism	Pneumocystis pneumonia	Cavitation; reticulonodular infiltrate; consolidation	Sputum	ITZ; AmB; PSZ	Cure

AmB, Amphotericin B; BAL, Bronchoalveolar lavage; CNS, central nervous system; COPD, chronic obstructive pulmonary disease; HIV, human immunodeficiency virus, ITZ, Itraconazole; TBF, Terbinafine; PSZ, Posaconazole. ^1^ Year of first isolation of *Sporothrix* spp. of pulmonary specimen. ^2^ T CD4+ cell count at the time of collection of pulmonary specimen.

## Data Availability

Not applicable.
